# On the feasibility of using TCR sequencing to follow a vaccination response – lessons learned

**DOI:** 10.3389/fimmu.2023.1210168

**Published:** 2023-07-13

**Authors:** Peter C. de Greef, Josien Lanfermeijer, Marion Hendriks, Alper Cevirgel, Martijn Vos, José A. M. Borghans, Debbie van Baarle, Rob J. de Boer

**Affiliations:** ^1^ Theoretical Biology and Bioinformatics, Utrecht University, Utrecht, Netherlands; ^2^ Center for Infectious Disease Control, National Institute for Public Health and the Environment, Bilthoven, Netherlands; ^3^ Center for Translational Immunology, University Medical Center Utrecht, Utrecht, Netherlands

**Keywords:** TCR - T cell receptor, pneumococcal 13-valent polysaccharide vaccine, immune response, high-throughput sequencing, vaccination

## Abstract

T cells recognize pathogens by their highly specific T-cell receptor (TCR), which can bind small fragments of an antigen presented on the Major Histocompatibility Complex (MHC). Antigens that are provided through vaccination cause specific T cells to respond by expanding and forming specific memory to combat a future infection. Quantification of this T-cell response could improve vaccine monitoring or identify individuals with a reduced ability to respond to a vaccination. In this proof-of-concept study we use longitudinal sequencing of the TCRβ repertoire to quantify the response in the CD4+ memory T-cell pool upon pneumococcal conjugate vaccination. This comes with several challenges owing to the enormous size and diversity of the T-cell pool, the limited frequency of vaccine-specific TCRs in the total repertoire, and the variation in sample size and quality. We defined quantitative requirements to classify T-cell expansions and identified critical parameters that aid in reliable analysis of the data. In the context of pneumococcal conjugate vaccination, we were able to detect robust T-cell expansions in a minority of the donors, which suggests that the T-cell response against the conjugate in the pneumococcal vaccine is small and/or very broad. These results indicate that there is still a long way to go before TCR sequencing can be reliably used as a personal biomarker for vaccine-induced protection. Nevertheless, this study highlights the importance of having multiple samples containing sufficient T-cell numbers, which will support future studies that characterize T-cell responses using longitudinal TCR sequencing.

## Introduction

Vaccination has proven to be a safe and effective method for immunization, limiting the spread of numerous infectious diseases. Exposure of a pathogen or its subunits to the adaptive immune system provides immunity that can potentially last a lifetime. Neutralizing antibody titers typically serve as a correlate of protection against infection in an individual (1–3) but do not cover the immunity provided by T cells, which is often crucial to prevent disease/severity of infection (4, 5). Quantitative characterization of the T-cell response induced by vaccination has the potential to provide an important additional measure of protection in an individual (6). T cells recognize antigens by their highly specific T-cell receptor (TCR) presented as peptides on the Major Histocompatibility Complex (pMHC). Activation through the TCR is followed by clonal expansion and maintenance at increased frequencies as memory T cells, resulting in an enhanced immune response at a next encounter with a similar pathogen. After vaccination, a T-cell response will be induced by the vaccine antigen. The TCR repertoire dynamics reflecting this response can be followed using high-throughput TCR repertoire sequencing (6–10).

Previous studies have used TCR repertoire sequencing to characterize the T-cell response after yellow fever vaccination (YFV) (7, 11). This live-attenuated virus vaccine induces a large CD8+ T-cell response, which could be quantified by measuring T-cell expansion and contraction after vaccination by longitudinally sequencing the TCR repertoire. This allowed the identification of YFV-specific TCR sequences, which occupied up to 8% of the total CD8+ T-cell repertoire two weeks after vaccination (7). Other vaccine-induced T-cell responses have been characterized by sequencing the TCR repertoire of cells that were sorted for binding known influenza epitopes (12, 13). For many other vaccines, however, the epitopes that induce a T-cell response remain unknown. Following vaccine-specific T-cell clones therefore requires characterization of the total TCR repertoire. In addition, responses can be conferred by either CD4+ or CD8+ T cells, which have different expansion dynamics, and occur at specific anatomical locations from which samples cannot be easily taken. It remains to be determined whether TCR sequencing of the overall (CD4/CD8) T-cell repertoire in blood can serve as a suitable biomarker to quantify T-cell responses induced by such vaccinations.

In the present proof-of-concept study we aimed to identify specific expansion of T-cell clones in the CD4+ memory T-cell pool after pneumococcal conjugate vaccination. Although this vaccine mainly induces pneumococcal serotype-specific antibodies, T cells are activated by the CRM197 conjugate, a carrier protein which is a non-toxic mutant of diphtheria toxin. The activated CD4+ T cells provide additional help to B cells to produce specific antibodies (14). As CRM197 is also the main antigen of the diphtheria vaccine given in early childhood, the vaccine is anticipated to boost existing T-cell memory. However, the height of the T-cell response may be lower compared to the T-cell response against YFV and immunodominant epitopes are less well described. We performed longitudinal TCR sequencing of the CD4+ memory T-cell pool in the blood before and after pneumococcal conjugate vaccination. By taking replicate samples, we defined quantitative requirements to classify expansions and we identified critical parameters that aid in reliable analysis of the data. The absence of detectable robust T-cell expansions in many of the vaccinated individuals illustrates the challenges of using TCR sequencing to quantify specific T-cell responses after vaccination. We conclude that the T-cell response induced by the conjugate in the pneumococcal vaccine is often too small or too diverse to allow for reliable quantification using TCR sequencing. Finally, our analysis identified specific requirements for monitoring T-cell responses using longitudinal TCR sequence data.

## Results

### Study design

**Figure 1:**
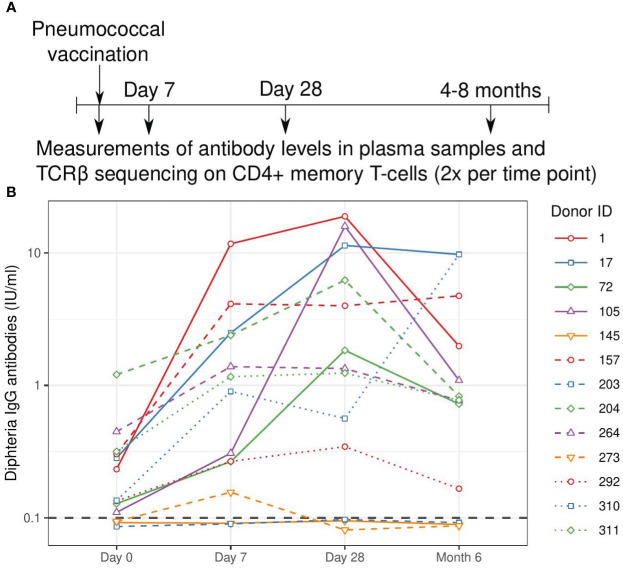
Study overview and measured antibody response. **(A)**. Schematic overview of the vaccination and sampling time course. Individuals were vaccinated at day 0, blood samples were drawn before vaccination (day 0) and at three follow-up time points. **(B)**. Quantification of the antibody response to the diphtheria toxin. Values above 0.1 IU/ml are considered protective and this threshold is indicated with a grey dashed line. Solid lines indicate older individuals (over 65 years of age), see **Table S1**.

We tested the application of TCRβ sequencing using samples from a human cohort that was part of a vaccination study with Prevanar 13, a conjugated vaccine targeting 13 pneumococcal strains. Blood samples were used from 13 adult individuals before vaccination (day 0) and at day 7, day 28, and between 4 to 8 months after vaccination (**Table S1**, **Figure 1A**). The antibody response was quantified by measuring diphtheria-specific IgG antibodies, which showed a clear response in 10 out of 13 individuals (**Figure 1B**). Typically, diphtheria-specific IgG levels were already at levels that are considered protective before vaccination, and increased about one order of magnitude at day 7 and/or day 28.

### The T-cell response can be characterized using longitudinal TCR repertoire sequencing

The presence of a clear antibody response after vaccination in most individuals suggests effective T-cell help, most likely provided by CD4+ memory T cells. We characterized this T-cell response by combining three subsets of CD4+ memory T cells (CD27^+^CD45RO^+^, CD27^-^/CD45RO^+^, and CD27^-^CD45RO^-^). The combined CD4+ memory T-cell populations were split in two portions, yielding sorted populations containing in the order of 10^5^ CD4+ memory T cells per subsample, per time point per individual (**Figure S1A**). mRNA was extracted from the cells in each sample for TCRβ cDNA library preparation (see Methods). The libraries were barcoded with Unique Molecular Identifiers (UMIs) to overcome biases in PCR amplification and to allow for error correction of the sequence reads.

**Figure 2:**
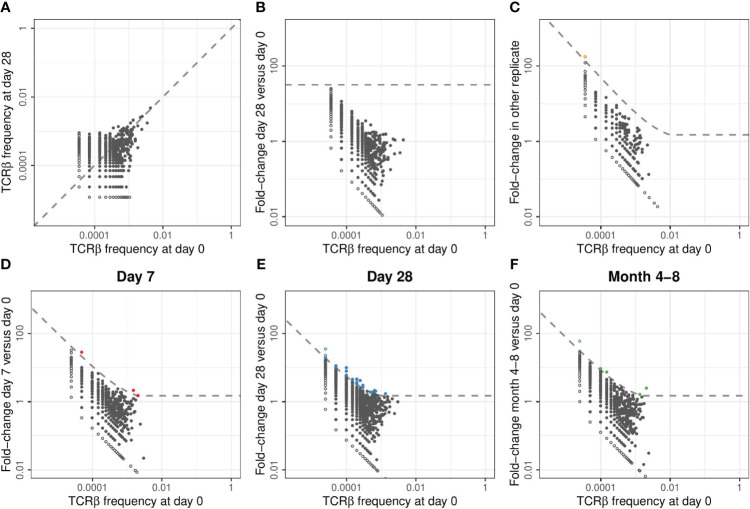
Classification of expansion between time points for TCRβ repertoires of example donor 17. **(A)**. Relative frequency of TCRβ sequences found at day 0 and/or day 28 in samples taken from donor 17. In each panel of this figure, the sequences that are observed at only one of the two time points are plotted as open symbols. For plotting and calculating the fold-change, we assigned these unobserved sequences half the frequency corresponding to a single UMI in the corresponding sample. In each panel, this results in the open dots appearing as two lines, at the left and the bottom, representing the sequences that were unobserved in either of the samples. The dashed line is the diagonal, representing equal frequencies in both samples. **(B)**. Fold-change in TCRβ frequencies observed in samples from donor 17 at day 28 versus day 0. The dashed line indicates a general fold-change threshold of 32x, as was used in (7), with no sequences exceeding this fold-change threshold. **(C)**. Fold-change in TCRβ frequencies between the two replicate samples taken at day 0 from donor 17. The dashed line represents the combined threshold for quantification of expansion (see Methods). Colored dots represent sequences that meet both requirements to be classified as expanded. In this case, comparing two samples from the same TCR repertoire, only a single sequence was classified as expanded (false-positive). **(D-F)**. Similar to **(C)**, but now quantifying the fold-change between different time points. Note that the dashed line defining the threshold for quantification of expansion differs between the panels because it is based on a combination of an absolute and relative change in frequency (see Methods). Sequencing data from replicate samples from each time point is joined, after which expansion at day 7 **(D)**, day 28 **(E)**, and month 4-8 **(F)** is quantified versus day 0.

Without prior knowledge which TCRs are induced by the conjugate of the pneumococcal vaccine, we relied on detection of expansion of TCRβ chains upon vaccination. One would expect the frequency of specific TCRβs to have increased at day 7 and/or 28 with respect to the pre-vaccination sample and potentially to be lower in the samples of the last time point. We thus measured the frequency of TCRβ sequences post-vaccination and compared these to the corresponding pre-vaccination frequencies (**Figure 2A**). TCRβ frequencies appeared highly correlated between time points and showed the persistence of many T-cell clones at relatively constant frequencies during the study period. We quantified the fold-change of each observed TCRβ sequence between pre-vaccination and post-vaccination time points, revealing the highest fold changes for the least abundant sequences (**Figure 2B**). Naturally these small clones give the strongest signal, as the fold-change results from dividing by a small pre-vaccination frequency. As a result, applying a general fold-change threshold to classify TCR sequences as being expanded would focus the analysis on those sequences of which the dynamics are estimated with the highest uncertainty (**Figure 2B**). A similar pattern (even) occurs when comparing two replicates from the same time point (**Figure 2C**), although these are samples containing cells from the exact same TCR repertoire.

As an alternative to a generic fold-change threshold, we used the many replicates within our dataset to estimate the effects of sampling noise during the generation, sequencing, and annotation of the TCRβ libraries (see Methods). We identified two requirements that together identify expanded TCRβs in our dataset: (1) a fold-change of at least 1.5, and (2) an absolute TCRβ-UMI count exceeding the relative pre-vaccination frequency by at least 30 UMIs. These thresholds were calibrated by balancing specificity, removing false-positive ‘expansions’ between samples from the same time point, and sensitivity, to allow for detection of expanded clones between time points (**Figure S2**). The combination of these requirements provides a fold-change threshold that is dependent on the pre-vaccination frequency and the size of the samples that are being compared. We classified few false-positive ‘expansions’ between samples from the same time point (orange point in **Figure 2C**), and a variable number of expanded TCRβs at the three post-vaccination time points (**Figures 2D-F**). To reduce the number of comparisons, while increasing the size of the samples being compared, we pooled the sequencing data from replicates covering the same time point when classifying expansion between time points before and after vaccination.

### Most repertoires allow for detection of few TCRβs that expand upon vaccination

**Figure 3:**
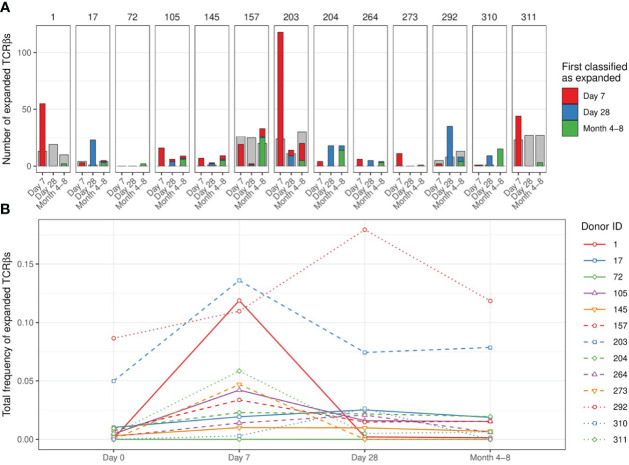
Number and dynamics of TCRβs sequences classified as expanded. **(A)**. Number of expanded TCRβs at time points after vaccination compared to day 0. Colors indicate the first time point at which the specific sequence was classified as expanded (red: day 7, blue: day 28, green: month 4-8). The classification of expansion was performed after pooling the replicates per time point (see **Figure S4A** for the classification based on comparisons of individual replicates between time points). The grey bars serve as a proxy for dynamics that are not induced by the vaccination, by classifying ‘expansion’ while permuting the post-vaccination and pre-vaccination time points. Specifically, we classified how many sequences would be considered ‘expanded’ in the pooled pre-vaccination samples, when compared to the indicated post-vaccination time points. **(B)**. Dynamics of the sequences classified as expanded at day 7 and/or day 28: the total relative frequency of these sequences is shown. Solid lines indicate older individuals (over 65 years of age).

We quantified TCRβ expansion after vaccination in each donor by applying the two requirements defined above when making pairwise comparisons between TCRβ frequencies at the pre-vaccination and the corresponding post-vaccination time points. In donor 203, for which we retrieved the largest number of TCRβ sequences (**Figure S1B**), this allowed us to identify > 100 TCRβ sequences that were expanded at day 7 and/or day 28 after vaccination (**Figure 3A**). These sequences together increased from about 5% of the repertoire to over 13% at the peak (day 7), followed by a decline to about 8% of the CD4+ memory pool by day 28 and month 4-8 after vaccination (**Figure 3B** – blue dashed line). The expansion of cells with these TCRβ sequences was reflected by a decrease in the size-normalized TCRβ diversity in the samples at day 7 post vaccination (blue dashed lines in **Figures S3D-F**). Strikingly, there was only little overlap between the TCRβs classified as expanded at day 7 and at day 28. In addition, while showing the largest number of expanded TCRβ sequences, donor 203 did not show a Diphtheria-specific IgG response after vaccination (blue dashed line in **Figure 1B**). These two observations together raise the question whether the detected expansions in this donor truly reflect the dynamics of a T-cell response induced by the vaccine. As we rely on the dynamics of the overall CD4+ memory T-cell repertoire, we cannot exclude the possibility that these expansions may have been caused by other ongoing immune responses. For example, this could be due to bystander activation of T cells that are not specific to an antigen in the vaccine. An alternative scenario is a response against CMV, as this donor turned out to be one of the only two CMV-positive individuals in our study (**Table S1**). Thus, although our findings suggest that longitudinal TCRβ sequencing can be used to detect T-cell clones that change in abundance after vaccination, they are not guaranteed to be specifically activated by the vaccine.

When investigating the smaller samples of the other donors we were unable to detect responses of a similar magnitude as in donor 203 using the same classification method. In all but one donor we detected TCRβ expansions at day 7 and/or day 28, although the expansions were again rarely detected at consecutive time points (**Figure 3A**). Donors 1 and 311 showed the largest number of expanded TCRβ sequences at day 7, while most expansions for donors 17 and 292 were detected at day 28 post vaccination. In some donors we identified the largest number of expansions at the latest time point (4-8 months), suggesting that not all expansions were induced by the vaccination. Our study did not involve TCRβ sequencing of individuals that did not receive the vaccination, which would have allowed us to estimate how many of the observed expansions were actually induced by the vaccination. To still validate our findings to some extent, we performed a permutation analysis by switching the order of the time points in each comparison. If we find a much larger number of expanded TCRβs before than after permutation, it suggests that the vaccination induced many of these expansions. In fact, the number of identified TCRβ expansions often did not exceed the number of ‘expansions’ after permutation, which in fact reflect contractions of a similar magnitude, for example by non-specific dilution (**Figure 3A** - grey bars). This indicates that many of the detected expansions may have occurred independently of the vaccination.

The total proportion of expanded TCRβs varied considerably between individuals, mostly owing to the different number of detected expansions for each donor (**Figure 3B**). The largest fraction of such sequences in the repertoire was observed in donors 203 and 292. Note that the total pre-vaccination frequency of these expanded sequences was also unexpectedly high (> 5%). This could indicate cross-reactivity of the T cells expressing these TCRβs but can also involve another immune response that was independent of the vaccination. We did not observe a clear relation between the breadth and/or depth of the identified response and the age of the donors. The largest contraction of expanded TCRβs happened between day 7 and day 28 post-vaccination in most individuals, most clearly pronounced in donors 1 and 203. Notably, detecting TCRβ expansions by making comparisons between individual replicates of different time points yielded similar numbers of expanded TCRβ sequences (**Figure S4A**). Thus, in most donors we only detected a few TCRβs that expanded upon vaccination at a single time point when compared to their pre-vaccination frequency.

### The sample size dominates the number of detected TCRβ expansions

The large differences that we observed in the number of expanded TCRβs between donor 203 and the other donors could be caused by a biological effect, but also by technical variation, e.g., the number of identified TCRβ sequences. In order to distinguish between biological variation regarding the vaccination response in different donors, and sources of technical variation between samples, we computationally down-sampled the TCRβ repertoires from donor 203 to the sample sizes of the corresponding samples from the other donors. In each of these down-sampled sets we could still detect expansions, but the number of identified TCRβ expansions was at least 5-fold lower for each down-sampled repertoire (**Figure S4B**). This emphasizes that the total number of identified TCRβ sequences is a critical parameter for longitudinal characterization of T-cell dynamics and a large determinant for the ability to detect expansions. This analysis also suggests that we might in fact have identified more TCRβ expansions in the other donors than we detected in donor 203, if their sample sizes would have been as large as those of donor 203 (**Figure S4B**).

The analysis so far focused on the identification of individual TCRβ sequences that expand upon vaccination. We also assessed whether the graph structure of the repertoire could be used to identify a robust TCR response. Classifying the expansion of clusters of similar TCRβ sequences yielded very similar results to the analysis of individual sequences (**Figure S5**). This indicates that the sampling depth is a limiting factor for identification of expansions, even at the level of clustered TCRβ sequences. We also checked if we could detect more general diversity signatures of TCR repertoire dynamics after vaccination. We quantified the changes in overall diversity of the TCRβ repertoire using various estimates (see Methods). The overall repertoire diversity varied considerably, but not consistently, between donors and time points (**Figures S3A-C**). Since the diversity measures are strongly affected by the sample size, we also normalized these estimates by down-sampling each repertoire to the same number of UMIs (**Figures S3D-F**). Even after size-normalization, we observed increases as well as decreases in TCRβ diversity upon vaccination. Even though the expansion of specific TCRβ sequences could be reflected in a decreased diversity after vaccination, we did not detect such dynamics consistently in most donors. An alternative scenario that has been proposed is that the recruitment of new (naive) vaccine-specific clones could increase the diversity of the TCR repertoire over time (9). The combination of these two opposite effects of vaccination on TCR diversity may explain why we do not detect consistent diversity dynamics between vaccinated individuals.

Another approach to follow the dynamics of many T-cell clones together is by quantifying the changes in TCRβ V-gene usage. The observation that TRBV usage varies considerably between samples, both from the same and from different time points (**Figure S6**), confirms that sampling effects have a profound effect on TCRβ frequencies, partly masking clonal dynamics that allow for quantification of the T-cell response induced by vaccination. Together, these results identify the size of T-cell samples as a key factor that determines to which extent T-cell responses can be quantified and compared using TCR sequencing.

**BOX 1 Challenges when following the vaccination response using longitudinal TCRβ sequences**

Its enormous diversity is one of the key features of the TCR repertoire, but also poses a major challenge to measure T-cell responses using longitudinal TCRβ sequencing. Without a priori knowledge about which TCRs are antigen-specific, responding T-cell clones must be distinguished from T-cell clones with different specificities, merely based on changes in their abundance. Thus, a sufficient increase in frequency is required to identify the potentially many TCRβ chains that are expressed by the cells mounting the antigen-specific T-cell response. For each involved T-cell clone, this requires: (1) an abundance in the repertoire that is sufficient to be present in the sample, (2) a TCR sequencing protocol that is sensitive enough to detect changes in frequency, and (3) a careful analysis to distinguish between technical variation and true clonal dynamics.

**1.** Sufficient abundance of cells of interest in the sorted cell population

**Figure 4:**
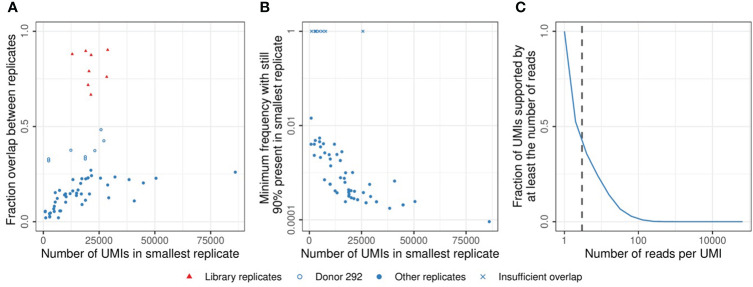
Sample overlap and coverage. **(A)**. Fraction of the sample overlapping between two replicates from the same time point (see Methods). Comparisons between multiple samples of sorted cells (blue circles) and replicates generated by sequencing the same TCRβ library twice (red triangles). The open blue circles indicate samples from donor 292, which have a relatively high overlap due to a low TCRβ diversity (see also **Figure S3**). **(B)**. TCRβ frequency in the largest replicate at which sequences start missing in the smallest replicate (see Methods). X marks indicate comparisons in which the most abundant TCRβ sequence did not overlap between both samples. **(C)**. Cumulative frequency of the UMI-coverage, plotted as the fraction of UMIs supported by at least a given number of reads (horizontal axis). The vertical dashed line indicates a coverage of 3 reads per UMI, which was used as the minimum support to take a sequence into account in the analysis (see Methods).


There are about 10^12^ T cells in the human body, so even samples of millions of T cells will only constitute a tiny proportion of the total T-cell pool. Combined with the large diversity of the TCR repertoire, this results in limited TCR overlap between samples of naive and memory T cells, even between replicates of the same time point (15). The measured overlap correlates strongly with the sequencing depth of the sample, which depends on the starting number of cells and the sequencing protocol (**Figure 4A**). These observations follow from the probability of clonal presence in a sample, which, for small samples, scales roughly linearly with the sample size. A central question is which proportion of the cells in the sample are expected to be participating in the response towards the vaccine. A previous study estimated the response after yellow fever vaccination (YFV) to comprise 2-8% of the total T-cell pool in blood, which is composed of many clones which frequencies differ by several orders of magnitude (7). Since most vaccines are expected to induce a much smaller response than YFV, the frequency of most vaccine-specific TCRβs will be very low, even at the peak of the response. It may thus be useful to enrich the sample for T cells that participate in the response, to obtain enough signal. In this study, we sorted the CD4+ memory T-cell population because a response was anticipated to occur within this cell population. Although we were limited by the availability and size of the samples, further enrichment may be possible by sorting for activation markers and antigen-specificity using available tetramers when possible. While enrichment for cells with specific characteristics allows for quantitative estimates of the total T-cell response, the identification of individual clonal T-cell dynamics will become more complicated.

**2.** Sensitive TCR sequencing protocols

When calculating the expected effect size in a sample for a given number of sorted cells, it is important to take the loss of information during the TCR sequencing protocol into account. We estimated that probably less than 10% of the cells contributed one or more mRNA molecules to the eventual dataset after amplification, sequencing, and processing of the data (**Figure S7**). Moreover, some of the cells perhaps contributed multiple mRNA molecules, each labeled with a separate UMI sequence, adding to the uncertainty of estimating clonal abundance before and after vaccination. The frequencies of TCRβs in the data is distorted by many stochastic processes, including the sampling of cells and mRNA molecules, as well as the amplification and sequencing of transcripts. We quantified the contribution of these factors by comparing replicate samples from the same TCR repertoire. This revealed that typically a frequency of at least 0.1% of the memory T-cell repertoire is required to be stably present in multiple samples at our average sequencing depth (median value in **Figure 4B**). We also found considerable differences in TCRβ abundance between replicates, requiring us to set strict thresholds to discriminate T-cell expansion from technical variation. Note that sequencing the same TCRβ library twice yielded much more similar results, indicating that most uncertainty is introduced before the sequencing (**Figure 4A** - red points). Having multiple samples from the same TCR repertoire is essential to estimate the contribution of technical variation to the measured abundances. Specific algorithms exist to model the noise introduced during TCR sequencing and to discriminate this from true TCR dynamics (16, 17). Another factor to consider is the contribution of uneven PCR amplification. While UMIs are used to factor this out, the UMI-based error correction of the sequences requires multiple reads sharing their UMI. The wide distribution of the number of reads per UMI results from the uneven amplification by PCR and identifies a sufficient sequencing depth as a key requirement to allow enough sequences to reach the threshold for error correction (**Figure 4C**).

**3.** Processing the samples and quantification of expansion

During the steps outlined above, from reverse transcription, via amplification, to sequencing the TCRβ libraries, it is inevitable that errors are introduced. As UMIs label cDNA molecules before amplification, they greatly assist in error-correction of the reads. Dedicated pipelines exist to perform these steps, which can also correct other errors by clustering of low-quality or nearby sequences (18, 19). The resulting data provides a way to estimate the changes in frequency of each TCRβ sequence. Careful interpretation is necessary for the reasons explained above, in order to distinguish between real biological effects and the technical variation arising during the entire TCR sequencing process. This requires a robust classification of expansion, which is ideally calibrated on multiple samples from the same and different time points.

## Discussion

In this proof-of-concept study, we applied longitudinal TCRβ sequencing on CD4+ memory T cells from individuals before and after pneumococcal conjugate vaccination. We developed specific criteria to classify clonal expansions from longitudinal TCR sequencing data, aiming to discriminate between biological and technical variation. By doing so, we identified some TCRβs that expanded after vaccination, although these were mostly limited to a few individuals and a single time point. The absence of detectable and persistent T-cell expansions in most individuals illustrates the complications of longitudinal TCR sequencing when there is a small and/or diverse T-cell response. The sample size appears a crucial factor for detection of TCRβ expansion in the overall T-cell repertoire. An overview of critical technical requirements for a robust longitudinal TCRβ repertoire characterization are detailed in **Box 1**.

The failure to detect considerable T-cell expansions upon pneumococcal conjugate vaccination does not imply absence of a substantial T-cell response, or lack of a protective effect of vaccination. Firstly, the total size of the T-cell response in these donors is unknown and may well be below the 1% range needed for TCR analysis at the current sampling depth. This means that even all vaccine-specific TCRβ sequences together may be relatively rare and not easily distinguishable from all TCRβ sequences with different antigen-specificities. Secondly, the total response is expected to be composed of many individual TCRαβ clonotypes with different TCRβ sequences. Many of their frequencies will fall below our limit of detection (**Figure 4B**) especially when we track cells at the level of individual TCRβ sequences. Moreover, even when TCRβ sequences are present at multiple time points, their dynamics cannot always be distinguished from noise. Thirdly, it is possible that the absence of a strong immune signal in samples obtained from blood is due to the fact that the activation and proliferation of vaccine-specific T cells occurs in lymphoid organs or specific tissues (20). Lastly, it remains to be determined whether the size and/or diversity of the T-cell response correlates with protection against infection. The sharp increase in Diphtheria-specific IgG antibodies indicates a substantial response upon vaccination, while the size and breadth of the T-cell response currently remain unclear as a functional characterization of the T-cell response was not included in this study.

TCR repertoire characterization is usually done by sequencing of the mRNA coding for the α- and/or β-chain of the TCR. While single-cell techniques exist to perform paired sequencing of both TCR chains, their drawback is the limited number of cells that can currently be profiled. Instead, many studies focus on the TCRβ-chain, for which high-throughput methods allow characterization of millions of cells. Our choice to sequence the bulk TCRβ repertoire instead of paired TCRαβ single-cell sequencing has both advantages and drawbacks. This way, we could characterize the TCR repertoire from many cells per time point, which is necessary to detect clonal expansions. Although missing information on the TCRα, a substantial expansion of a TCRαb clonotype will likely be reflected by an increased frequency of its TCRβ sequence in the total T-cell pool. More detailed identification of the expanded TCRs in donor 203 would have required single-cell analysis of many cells. This could have allowed us to further characterize the expanded clones, for example by analyzing their transcriptional profiles. In addition, the technical variation stemming from the fact that cells can contribute multiple mRNA molecules in a bulk analysis is excluded when sequencing the repertoire at the single-cell level. Currently, however, considering the limited expansion observed in most bulk repertoires, we do not expect that we could have captured a larger response using single-cell sequencing, because sample sizes would have been even smaller. Single-cell TCR sequencing is therefore a more promising avenue to analyze vaccinations for which tetramers are available to enrich the sorted population for antigen-specific T cells.

A key challenge remains to distinguish between technical variation and true dynamics of the TCR repertoire. This discrimination requires sufficiently large sample sizes, which in turn requires large numbers of input cells and minimization of information loss during the TCR-sequencing procedure (see also **Box 1**). This reduces the relative contribution of sampling noise and technical biases, which allows setting less strict thresholds to quantify expansion. The TCR dynamics result from a combination of vaccine-induced expansions and other ongoing immune responses. Thus, functional assays are crucial to verify the specificity of the expanded T-cell clones. Such information will also help to interpret the dynamics functionally, such as the changes in TCR diversity after vaccination. The large variation in MHC-genes across individuals causes immune responses to be mostly private. Still, finding motifs in vaccine-specific TCR sequences would enable more direct identification of the vaccine-induced T-cell response (21), perhaps even without the need for data from consecutive time points.

Some vaccines, like YFV, may elicit large T-cell responses that can be accurately quantified by longitudinal TCR sequencing of the whole T-cell pool. The effects of vaccines that activate fewer T cells, or a wide diversity of T-cell clones will be much more challenging to characterize using sequencing of the TCR repertoire. Translating parameters of the T-cell response into a personalized biomarker of vaccine efficacy involves several other challenges. A first step would be to relate these to other correlates of protection such as (neutralizing) antibody titers. For example, this may give insight into the importance of the breadth and depth of the T-cell response, which can be estimated using TCR sequencing. The relevance of such features beyond the currently known risk factors and serological assays will require extensive clinical studies. While currently perhaps not yet feasible, technological advances may enable this in the future. This study should be considered as one of the first steps on the way to personalized vaccination strategies that will further protect people at risk from infectious diseases.

## Materials and methods

### Study cohort

Samples used in this study were selected from The Vaccines and InfecTious disease in the Ageing PopuLation (VITAL) cohort (22), which was started in 2019 in the Netherlands. For this study, healthy individuals were recruited who did not use immune-modulatory drugs and who were not immunocompromised due to a medical condition. This study was approved by Medical Research Ethics Committee Utrecht (EudraCT Number: 2019–000836–24) (Anon Clinical Trials register) and carried out in accordance with the recommendations of Good Clinical Practice with written informed consent from all subjects, in accordance with the Declaration of Helsinki.

### Sample selection

For this study, the samples were selected from the VITAL cohort. We selected 8 donors with an age between 25 and 40 years (young adults), as well as 5 adults who were over 65 years (older adults). Individuals were vaccinated with the pneumococcal conjugate vaccine Prevenar 13. Blood samples were collected from all individuals at day 0 (before vaccination), at day 7, at day 28 and between 4 to 8 months post-vaccination (see **Table S1**).

### PBMC and serum isolation

Peripheral blood mononuclear cells were obtained by Lymphoprep (Progen) density gradient centrifugation from heparinized blood, according to the manufacturer’s instructions. PBMCs were frozen in 90% fetal calf serum and 10% dimethyl sulfoxide at -135°C until further use. Serum was isolated out of tubes with clot-activation factor and stored at -80°C until further use. Blood withdrawals were postponed if participants received other vaccinations or had elevated body temperatures (>38°C).

### Cytomegalovirus-specific antibodies

Anti-CMV IgG antibody concentrations were measured by an in-house-developed multiplex immunoassay (23). Cutoff values were based on previous calculations: individuals with a CMV-specific antibody level of ≤4 arbitrary units (RU)/ml were considered CMV-negative, individuals with an antibody level > 7.5 RU/ml were considered CMV-positive, and those with a level between 4 and 7.5 RU/ml were considered inconclusive and hence not selected for this study (24).

### Determination of diphtheria-specific antibody concentrations

Nunc MaxiSorp ELISA plates were coated with 2.5µg/ml diphtheria toxoid (Statens Serum Institute) and blocked with 0.01M Glycin. Plasma samples were analysed in duplicates. Bound antibodies were detected with HRP-conjugated secondary Rabbit Anti-Human IgG Antibody (Sigma-Aldrich) and TMB one component Substrate Solution (Diarect). IgG antibodies were quantified in IU/ml using Standard Diphtheria Antitoxin Human Serum (NIBSC). The detection limit of the assays used was 0.015 IU/ml and antibody concentrations above 0.1 IU/ml were considered as protective.

### Isolation of CD4+ memory T cells for TCR repertoire analysis

Previously frozen PBMCs were thawed at 37°C and washed once with RPMI 10% fetal calf serum and twice with RPMI 5% fetal calf serum for sorting. Approximately 5 million PBMCs were labeled at 4°C for 30 min with the following monoclonal antibody (mAbs) mix: CD8(RPTA-T8)-FITC, CD3(UCHT1)-PerCP, CD4(RPA-T4)-APC/Cyanine7, CD27(O323)-Brilliant Violet 765, CD45RO(UCHL1)-PE (All Biolegend). After the staining was finished, samples were split in two portions and sorted separately to obtain duplicates. CD4+ memory T cells were defined as CD27+/CD45RO+, CD27-/CD45RO+, or CD27-/CD45RO- (thus, all CD4+ cells, except the naive T cells (CD27+/CD45RO-)) and sorted using a FACS Melody cell sorter (BD). CD4+ memory T cells were sorted directly into PBS, spun down and resuspended in RNA Later (Ambion Inc. Applied Biosystems). Sorted samples were stored at −80°C for subsequent TCRβ clonotype analysis.

### Preparation of TCRβ cDNA libraries for sequencing

mRNA was isolated with the RNA microkit (Qiagen) according to the manufacturer’s protocol. Isolated mRNA was used in the 5’ RACE-based SMARTer Human TCR a/b Profiling Kit v2 (Takara Bio USA, Inc.) to perform sequencing of TCRs, following the manufacturer’s protocol using only the TCRβ-specific primers. Cleanup was performed with AMPURE XP clean-up beads (BD). The resulting TCRβ libraries were sequenced on an Illumina MiSeq sequencer (paired-end 2x300nt). The reproducibility of the sequencing was analyzed by sequencing the libraries of donor 145 twice. A larger number of shorter reads was obtained for donor 204 and 292 on an Illumina NextSeq sequencer (paired-end 2x150nt) instead, although this did not lead to a dramatic increase in identified expansions (**Figure 3A**).

### Processing of TCRβ sequencing data

TCRβ sequencing data was processed using the Cogent™ NGS Immune Profiler pipeline (version 1.0), as provided by Takara Bio. We set the overseq-threshold to 3, meaning that UMI-TCR pairs supported by at least 3 reads were taken into account. We defined a TCRβ sequence as the combination of V-segment, CDR3 amino acid sequence and J-segment. For the analyses presented in **Figures 2D-F**, **Figure 3**, **Figure S3**, and **Figure S5** we joined the counts from replicates of the same time point to arrive at a single TCR repertoire per time point. The equivalent analysis of **Figure 3A** using the individual replicates is shown in **Figure S4A**.

### A robust classification of expansion

TCRβ sequences have frequencies that can differ by multiple orders of magnitude. The most abundant sequences are often measured with a sufficient number of UMIs to reliably estimate their frequency in the repertoire. The frequency of many rare sequences is much less certain, as their proportion in the data is relatively more affected by sampling noise. While classifying expansion between time points, we accounted for these differences using a two-step approach. We used replicates of the same time point of the same donor (which are thus samples from the same T-cell repertoire) to estimate the sampling noise. The requirements for expansion were optimized to be both specific and sensitive: they should result in little or no expansion between samples from the same time point, while allowing for detecting expansions between time points.

Firstly, we determined a general fold-change threshold based on the abundant TCRβ sequences. Specifically, we analyzed the sequences present at a relative frequency of more than 0.5% in a sample and quantified their fold-change when comparing with another sample taken. For sequences that were present in one sample, but absent in the other, the fold-change would result in division by zero. To still obtain a fold-change in such cases, we assigned these unobserved sequenced a frequency equal to the frequency of a single UMI in the corresponding sample, divided by two. We determined the optimal fold-change threshold by comparing replicates of the same time point, and samples from different time points. Since we know that, by definition, there should be no expansion between the repertoires sampled at the same time point, we used 1.5 as the optimal fold-change threshold for our samples (**Figure S2A**).

Secondly, we quantified the sampling noise, which is expected to have a larger effect on the fold-change of rare sequences. Based on the relative frequency of a sequence in a reference sample, we calculated its expected number of UMIs in another sample. If no further threshold would be added, this would result in many sequences to be classified as expanded, even between samples from the same time point (**Figure S2B**). We therefore added a threshold to this number (accounting for the contribution of sampling noise to the absolute UMI count), to obtain the minimum UMI count to be classified as expanded compared to the reference sample. By setting an absolute UMI count threshold of 30 UMIs, we decreased the number of expanded sequences between samples from the same time point, while still allowing the detection of expansions between time points (**Figures S2B, C**).

Thus, we classify a sequence as expanded between sample 1 and 2, if (1) the relative frequency in sample 2 is at least 1.5 times higher than in sample 1, and (2) the absolute UMI count in sample 2 exceeds the relative frequency in sample 1 with at least 30 UMIs. These two requirements together result in the dashed lines shown in **Figures 2C-F**. The formula for these lines is given by $FC=\text{max}\left(Th_{FC},\;1+\;\frac{Th_{UMI}}{f_{pre}\;\times \;N_{post}}\right)$ , in which FC is the fold change (as plotted on the vertical axis), $Th_{FC}$ is the general fold change threshold, $Th_{UMI}$ is the UMI threshold, $f_{pre}$ is the relative clonal frequency at the pre-vaccination time point (as plotted on the horizontal axis), and $N_{post}$ is the total number of UMIs measured at the post-vaccination time point.

### Quantification of overlap between samples

We quantified the TCRβ overlap between sample pairs using Bray-Curtis dissimilarity, because it takes abundance into account and its value can be intuitively understood. For a collection of TCRβ X with proportions ${\text{X}}_{\text{i}}$ and ${\text{X}}_{\text{j}}$ in sample i and j , respectively, the Bray-Curtis dissimilarity is calculated as $\text{BC}=1\;-\text{Σ min}\left({\text{X}}_{\text{i}},{\text{ X}}_{\text{j}}\right)$ . The relative overlap, 1 −BC , can thus be understood as the proportion that is identical between two samples, in terms of identity and abundance, such that no overlap remains if this part is removed from both samples.

To obtain a quantitative estimate of the minimum resolution of the TCRβ sequencing assay, we compared replicates from the same time point from the same individual with each other. Since these are obtained from the same TCR repertoire, they provide the opportunity to estimate the minimum TCRβ sequence frequency that guarantees overlap between samples. We then sorted the TCRβ sequences based on abundance in the largest sample. Starting from the sequence with the highest frequency, we kept track which fraction of the TCRβs was also observed in the smaller replicate. Continuing this until less than 90% of the most abundant sequences overlapped, we obtained an estimate on the minimum TCRβ frequency that is required to guarantee overlap between samples from the same TCR repertoire.

### Diversity estimates

Many measures exist to quantify diversity of a sample, which mostly differ in the relative contribution of richness and evenness. Richness relates to the distinct number of TCRβ sequences in a sample, while evenness quantifies the differences in abundance between TCRβ sequences. We used three distinct measures to estimate the TCRβ diversity in our samples. The richness is the total number of distinct TCRβ sequences in the sample. Given a collection of TCRβ X with proportions ${\text{X}}_{\text{i}}$ in a sample, the Shannon index is $\text{H}=\;-{\text{Σ X}}_{\text{i }}{\text{ lnX}}_{\text{i}}$ , which can be expressed as the effective number of species by ${\text{e}}^{\text{H}}$ . The Simpson index is given by $\text{λ}={\text{ Σ X}}_{\text{i}}^{2}$ , of which the inverse 1 /λ is the effective number of species. These three measures were evaluated for the TCRβ repertoires at each time point, and plotted in **Figures S3A-C**. To compare diversity between donors and time points while accounting for the different sample sizes, we computationally down-sampled all samples to have a total number of UMIs equal to the smallest sample in the set. The normalized diversity measures calculated from these down-sampled repertoires are provided in **Figures S3D-F**.

## Data availability statement

The original contributions presented in the study are publicly available. This data can be found here: https://www.ncbi.nlm.nih.gov/bioproject/PRJNA975568 (NIH Sequence Read Archive (SRA) Bioproject PRJNA975568).

## Ethics statement

The studies involving human participants were reviewed and approved by Medical Research Ethics Committee Utrecht (EudraCT Number: 2019–000836–24) (Anon Clinical Trials register). The patients/participants provided their written informed consent to participate in this study.

## Author contributions

PG, JL, JB, DB, and RB conceived the study. The experimental procedures were carried out by JL, MH, AC, and MV. PG performed the data analysis in consultation with the other authors. PG and JL wrote the manuscript with input from JB, DB, and RB. The manuscript was edited and approved by all authors.

## Supplementary material

Supplementary Figure 1 Number of sorted cells and identified TCRβ sequences. **(A)**. Number of sorted CD4+ memory T cells per sample. While the numbers of sorted cells for donor 203 were in the same range as the other samples, the exact numbers could not be retrieved, and are therefore not plotted. **(B)**. Total number of TCRβ sequences retrieved by TCR sequencing per sample.

Supplementary Figure 2 Thresholds for classification of expanded sequences. **(A).** The fraction of abundant sequences (> 0.5% in the reference sample) that would be classified as expanded because their fold-change exceeds the threshold (horizontal axis). Comparisons were made between samples from the same time point (blue) and between a reference sample and a sample from a later time point (red). The vertical dashed line indicates a fold-change of 1.5, which was used in the analysis to classify expansion. **(B)**. Similar to A, but now for all sequences and additionally requiring an absolute difference in UMI count. A sequence is classified as expanded if (1) the fold-change is larger than 1.5, and (2) the number of UMIs for that sequence exceeds the reference relative frequency with at least the excess UMI threshold (that varies on the horizontal axis). The vertical dashed line indicates the threshold of 30 UMIs, at which the fraction of sequences classified as expanded between time points is twice as high as within a single time point. This threshold was used in the analysis to classify expansion. **(C)**. Sensitivity analysis for the excess UMI threshold. Plotted is the number of expansions at post-vaccination time points (red circles: day 7, blue triangles: day 28, green squares: month 4-8) for each donor. The vertical axis has a logarithmic scale with 0 added below the horizontal dotted line to represent cases in which no expansions were identified.

Supplementary Figure 3 Estimated TCRβ diversity in pooled replicates before and after vaccination. **(A)**. Number of distinct TCRβ sequences per time point, after pooling the sequence counts of the corresponding replicates. The vertical axes have a logarithmic scale. **(B)**. Effective number of species (TCR sequences) as quantified with the Shannon index (see Methods). **(C)**. Effective number of species (TCR sequences) as quantified with the Simpson index (see Methods). **(D-F)**. Similar to A-C, but after normalizing the size of the samples by down-sampling to the smallest sample (2613 UMIs) to allow for comparison between time points and individuals.

Supplementary Figure 4 Expansion based on individual replicates and down-sampling of pooled samples. **(A)**. Similar to , for which samples from the same time point were pooled before classification of expansion. Here, the comparisons were made between individual replicates, meaning that a sequence will be classified as expanded if it satisfies both requirements in any of the post-vaccination replicates compared to any of the pre-vaccination replicates. The number of TCRβs meeting both requirements of expansion in any of those comparisons is plotted. Colors indicate the first time point at which the specific sequence was classified as expanded (red: day 7, blue: day 28, green: month 4-8). The grey bars serve as a proxy for dynamics that are not induced by the vaccination, by classifying ‘expansion’ while reversing the order of the time points. **(B)**. Comparison of the number of detected expansions in donor 203 versus the other donors, after correcting the data for the different sample sizes by down-sampling. We down-sampled the data of donor 203, pooled per time point like in , at each time point 10 times to the number of UMIs that were identified for the corresponding pooled samples from each of the other donors. Plotted on the vertical axis is the number of detected expansions for donor 203 that remain after down-sampling (median ± standard deviation), which is compared to the number of detected expansions of the indicated donor on the horizontal axis. The dashed line indicates the identity line, which indicates an identical number of detected expansions in both cases. The low numbers of expansions that remain after down-sampling indicate that the sample sizes of most of the donors were insufficient to detect the breadth of the response that was detected in donor 203. The observation that the other donors often showed more expanded clones than observed in the equally sized samples of donor 203, suggests that they may in fact have experienced a more diverse T-cell response than donor 203.

Supplementary Figure 5 Assessing expansions from the TCRβ graph structure. Number of expanded TCRβ clusters at time points after vaccination compared to day 0. Clusters were made by connecting TCRβ sequences, from all time points for a given individual, differing by a single amino acid substitution. Colors indicate the first time point at which the specific cluster was classified as expanded (red: day 0, blue: day 28, green: month 4-8) and color darkness represents cluster size (dark: > 1 TCRβ sequence, light: 1 TCRβ sequence). The classification of expansion was performed after pooling the replicates per time point. The grey bars serve as a proxy for dynamics that are not induced by the vaccination, by classifying ‘expansion’ while permuting the post-vaccination and pre-vaccination time points. Specifically, we classified how many clusters would be considered ‘expanded’ in the pooled pre-vaccination samples, when compared to the indicated post-vaccination time points.

Supplementary Figure 6 TRBV usage differences between samples from the same TCR repertoire and another time point. Comparison of TRBV usage between samples from the same individual. The total difference in TRBV usage between samples is quantified by summing the differences in relative frequency for each TRBV gene. Comparisons are performed per donor, between samples from the same time point (red dots) and different time points (blue triangles). The result of each comparison is plotted, with the boxes summarizing the median difference (thick line inside), as well as the first and third quartiles (bottom and top of the box, respectively).

Supplementary Figure 7 TCRβ contribution per input cell. **(A).** Total number of TCRβ mRNA molecules (uniquely labeled with UMIs) retrieved by TCR sequencing as a function of the number of sorted CD4+ memory T cells for the corresponding sample. **(B)**. Average mRNA contribution per input cell, as measured by dividing the total number of TCRβ sequences by the number of input cells. The horizontal dashed line shows the mean value. This can be considered an upper bound of the probability a given cell in the sample will contribute an mRNA molecule, as cells can contribute multiple mRNA molecules. **(C)**. Fraction overlap (see Methods) between replicates before and after redistributing sequences over the samples. The relative fraction of TCR sequences that overlap between the two replicates is shown on the horizontal axis. We then combined the TCRβ counts of both samples and randomly redistributed the sequences to arrive at two artificial samples with total counts identical to the original samples. We performed this redistribution 100 times, yielding 100 estimates for the overlap after redistribution. The median of these values is plotted on the vertical axis, with error bars indicating the standard deviation (often invisible due to the range of the error bars being smaller than the plot symbols). The dashed line indicates identical overlap between two samples for both comparisons, which is the expectation if every cell in the sample would have contributed maximally one mRNA molecule (25). The increase of overlap for all sample pairs after redistribution indicates that a substantial fraction of the cells contributed multiple mRNA molecules. Hence, the probability for a given cell to contribute a TCRβ mRNA is expected to be considerably lower than the upper bound shown in **(B)**.

Supplementary Table 1 Donor characteristics.
