# Supplementary figures and images for: On the feasibility of using TCR sequencing to follow a vaccination response – lessons learned
Source: Front Immunol. 2023 Jul 13;14:1210168. doi: 10.3389/fimmu.2023.1210168 (PMC10374308; doi:10.3389/fimmu.2023.1210168)

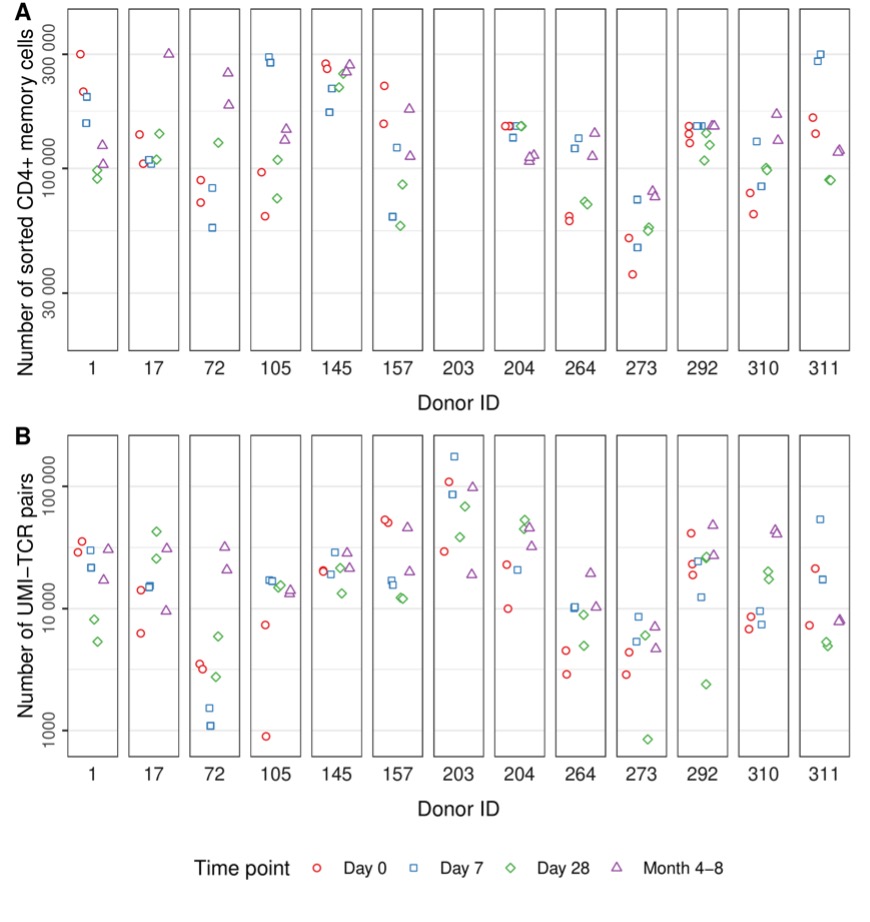

Supplement: Supplementary Figure 1 — Number of sorted cells and identified TCRβ sequences. (A). Number of sorted CD4+ memory T cells per sample. While the numbers of sorted cells for donor 203 were in the same range as the other samples, the exact numbers could not be retrieved, and are therefore not plotted. (B). Total number of TCRβ sequences retrieved by TCR sequencing per sample. [file Image_1.jpeg]

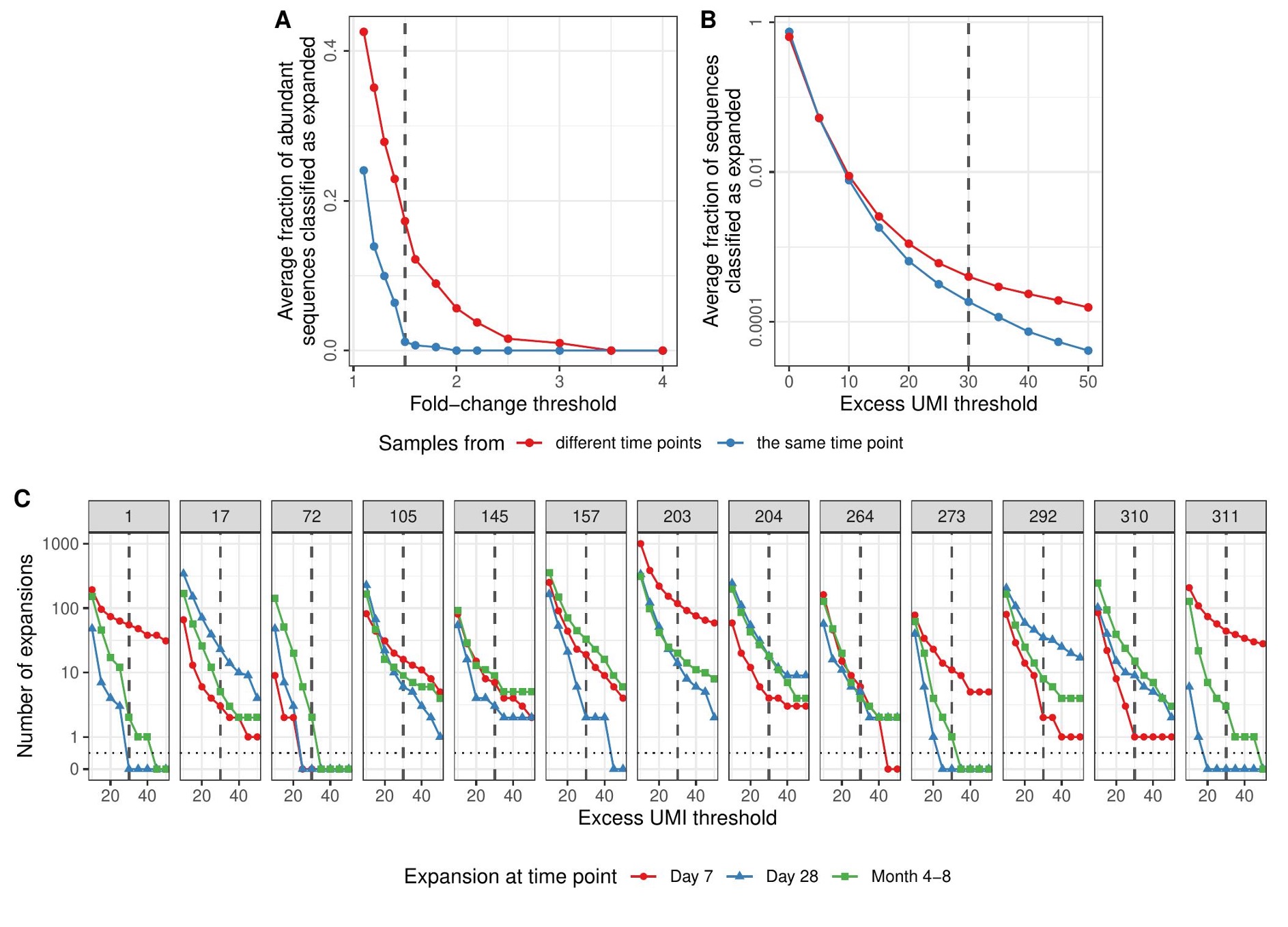

Supplement: Supplementary Figure 2 — Thresholds for classification of expanded sequences. (A). The fraction of abundant sequences (> 0.5% in the reference sample) that would be classified as expanded because their fold-change exceeds the threshold (horizontal axis). Comparisons were made between samples from the same time point (blue) and between a reference sample and a sample from a later time point (red). The vertical dashed line indicates a fold-change of 1.5, which was used in the analysis to classify expansion. (B). Similar to A, but now for all sequences and additionally requiring an absolute difference in UMI count. A sequence is classified as expanded if (1) the fold-change is larger than 1.5, and (2) the number of UMIs for that sequence exceeds the reference relative frequency with at least the excess UMI threshold (that varies on the horizontal axis). The vertical dashed line indicates the threshold of 30 UMIs, at which the fraction of sequences classified as expanded between time points is twice as high as within a single time point. This threshold was used in the analysis to classify expansion. (C). Sensitivity analysis for the excess UMI threshold. Plotted is the number of expansions at post-vaccination time points (red circles: day 7, blue triangles: day 28, green squares: month 4-8) for each donor. The vertical axis has a logarithmic scale with 0 added below the horizontal dotted line to represent cases in which no expansions were identified. [file Image_2.jpeg]

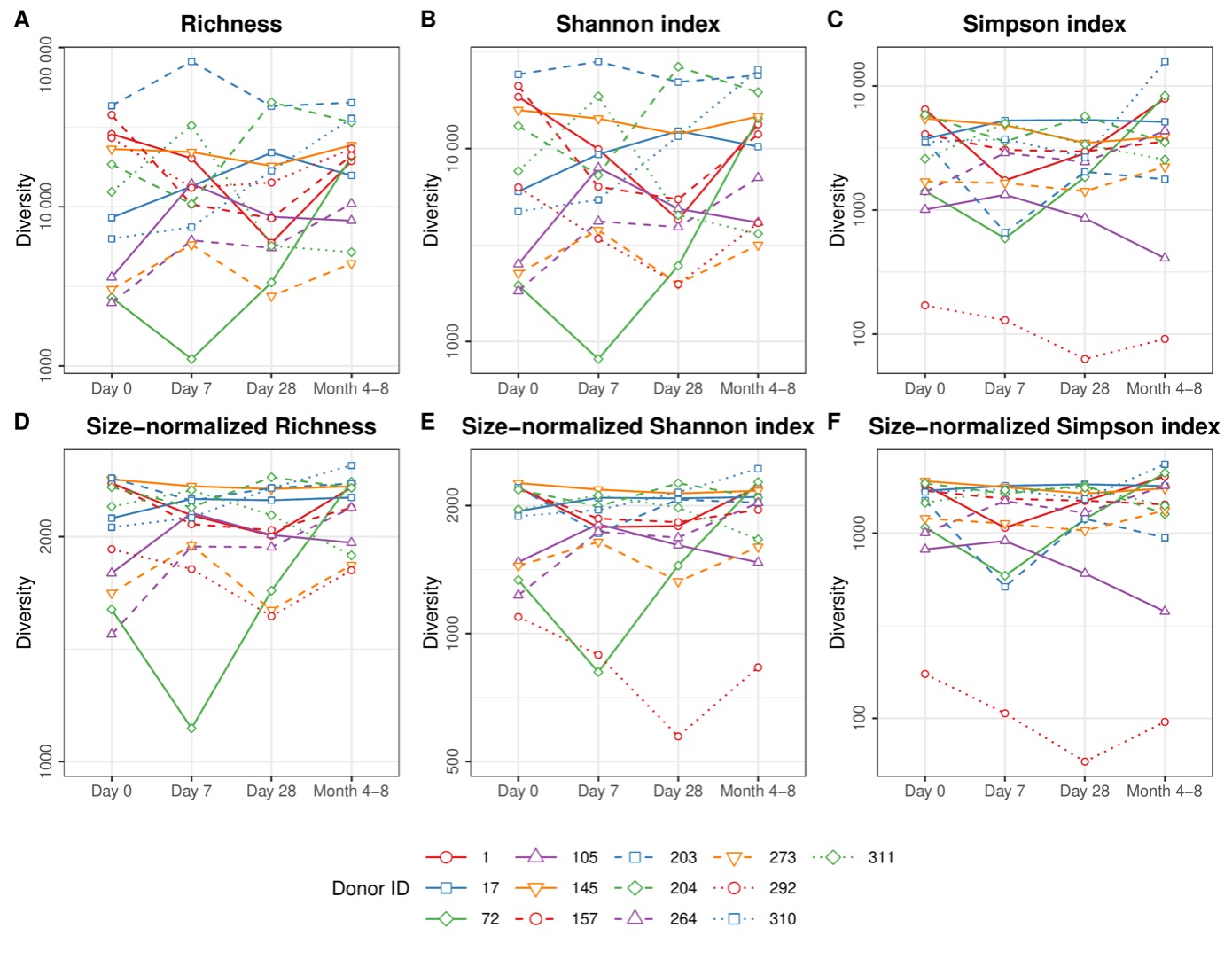

Supplement: Supplementary Figure 3 — Estimated TCRβ diversity in pooled replicates before and after vaccination. (A). Number of distinct TCRβ sequences per time point, after pooling the sequence counts of the corresponding replicates. The vertical axes have a logarithmic scale. (B). Effective number of species (TCR sequences) as quantified with the Shannon index (see Methods). (C). Effective number of species (TCR sequences) as quantified with the Simpson index (see Methods). (D-F). Similar to A-C, but after normalizing the size of the samples by down-sampling to the smallest sample (2613 UMIs) to allow for comparison between time points and individuals. [file Image_3.jpeg]

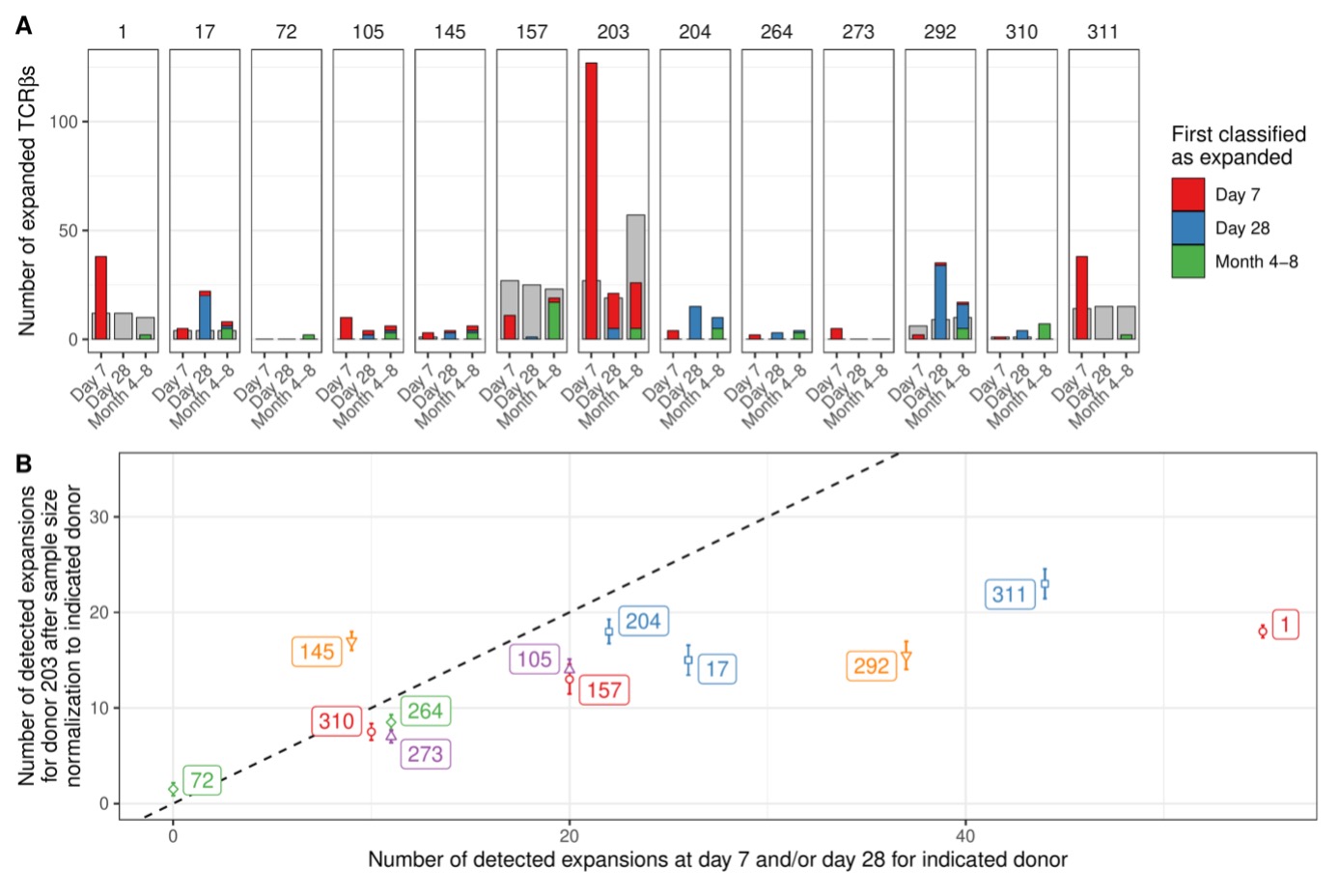

Supplement: Supplementary Figure 4 — Expansion based on individual replicates and down-sampling of pooled samples. (A). Similar to , for which samples from the same time point were pooled before classification of expansion. Here, the comparisons were made between individual replicates, meaning that a sequence will be classified as expanded if it satisfies both requirements in any of the post-vaccination replicates compared to any of the pre-vaccination replicates. The number of TCRβs meeting both requirements of expansion in any of those comparisons is plotted. Colors indicate the first time point at which the specific sequence was classified as expanded (red: day 7, blue: day 28, green: month 4-8). The grey bars serve as a proxy for dynamics that are not induced by the vaccination, by classifying ‘expansion’ while reversing the order of the time points. (B). Comparison of the number of detected expansions in donor 203 versus the other donors, after correcting the data for the different sample sizes by down-sampling. We down-sampled the data of donor 203, pooled per time point like in , at each time point 10 times to the number of UMIs that were identified for the corresponding pooled samples from each of the other donors. Plotted on the vertical axis is the number of detected expansions for donor 203 that remain after down-sampling (median ± standard deviation), which is compared to the number of detected expansions of the indicated donor on the horizontal axis. The dashed line indicates the identity line, which indicates an identical number of detected expansions in both cases. The low numbers of expansions that remain after down-sampling indicate that the sample sizes of most of the donors were insufficient to detect the breadth of the response that was detected in donor 203. The observation that the other donors often showed more expanded clones than observed in the equally sized samples of donor 203, suggests that they may in fact have experienced a more diverse T-cell response than donor 203. [file Image_4.jpeg]

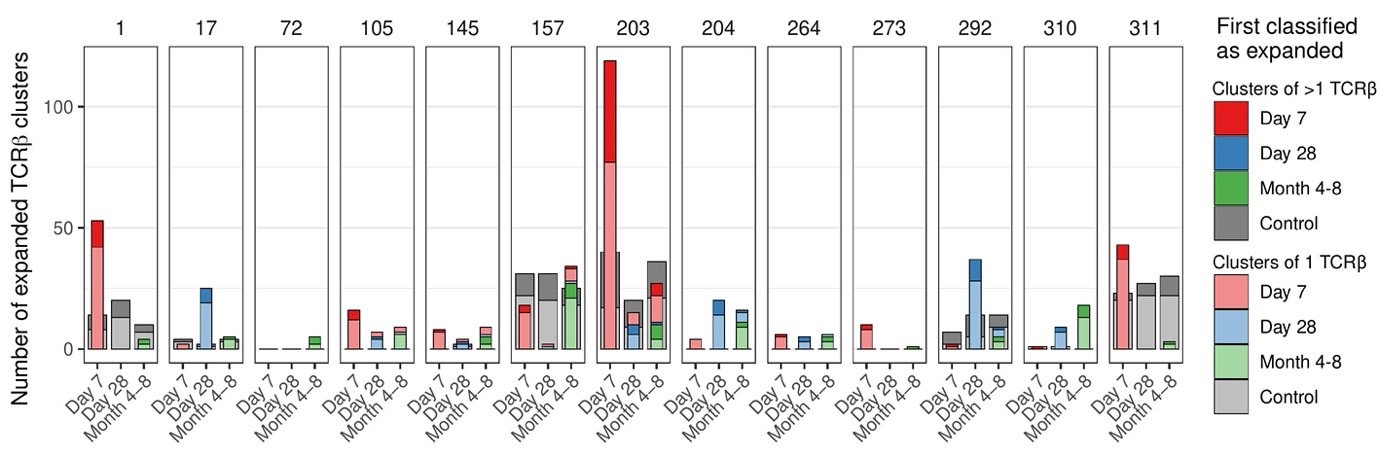

Supplement: Supplementary Figure 5 — Assessing expansions from the TCRβ graph structure. Number of expanded TCRβ clusters at time points after vaccination compared to day 0. Clusters were made by connecting TCRβ sequences, from all time points for a given individual, differing by a single amino acid substitution. Colors indicate the first time point at which the specific cluster was classified as expanded (red: day 0, blue: day 28, green: month 4-8) and color darkness represents cluster size (dark: > 1 TCRβ sequence, light: 1 TCRβ sequence). The classification of expansion was performed after pooling the replicates per time point. The grey bars serve as a proxy for dynamics that are not induced by the vaccination, by classifying ‘expansion’ while permuting the post-vaccination and pre-vaccination time points. Specifically, we classified how many clusters would be considered ‘expanded’ in the pooled pre-vaccination samples, when compared to the indicated post-vaccination time points. [file Image_5.jpeg]

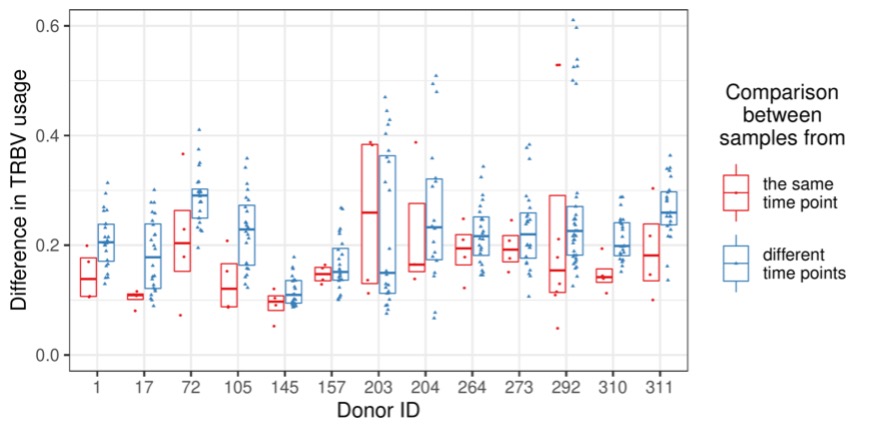

Supplement: Supplementary Figure 6 — TRBV usage differences between samples from the same TCR repertoire and another time point. Comparison of TRBV usage between samples from the same individual. The total difference in TRBV usage between samples is quantified by summing the differences in relative frequency for each TRBV gene. Comparisons are performed per donor, between samples from the same time point (red dots) and different time points (blue triangles). The result of each comparison is plotted, with the boxes summarizing the median difference (thick line inside), as well as the first and third quartiles (bottom and top of the box, respectively). [file Image_6.jpeg]

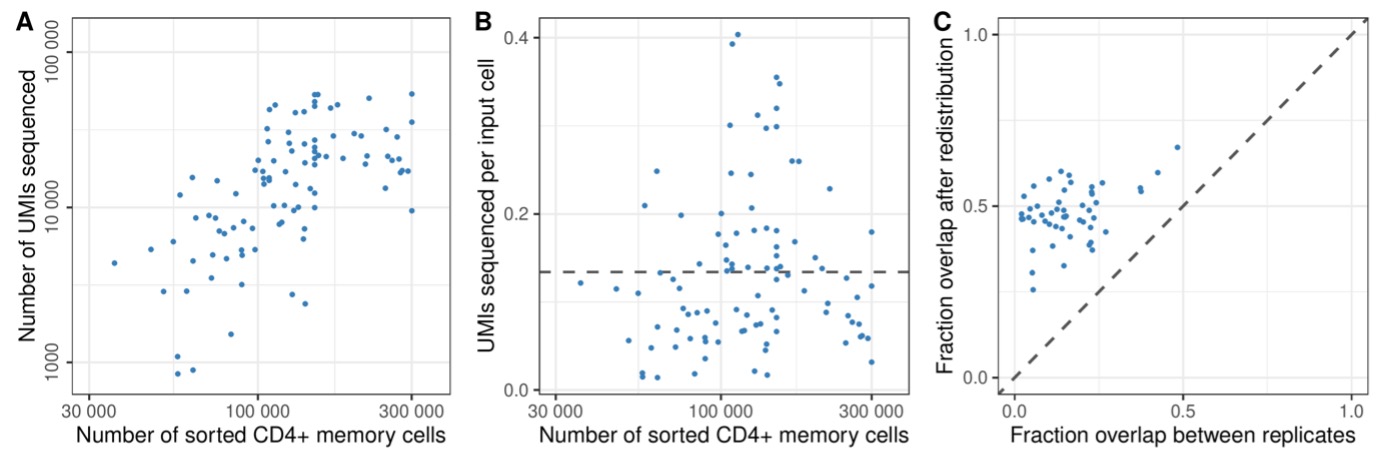

Supplement: Supplementary Figure 7 — TCRβ contribution per input cell. (A). Total number of TCRβ mRNA molecules (uniquely labeled with UMIs) retrieved by TCR sequencing as a function of the number of sorted CD4+ memory T cells for the corresponding sample. (B). Average mRNA contribution per input cell, as measured by dividing the total number of TCRβ sequences by the number of input cells. The horizontal dashed line shows the mean value. This can be considered an upper bound of the probability a given cell in the sample will contribute an mRNA molecule, as cells can contribute multiple mRNA molecules. (C). Fraction overlap (see Methods) between replicates before and after redistributing sequences over the samples. The relative fraction of TCR sequences that overlap between the two replicates is shown on the horizontal axis. We then combined the TCRβ counts of both samples and randomly redistributed the sequences to arrive at two artificial samples with total counts identical to the original samples. We performed this redistribution 100 times, yielding 100 estimates for the overlap after redistribution. The median of these values is plotted on the vertical axis, with error bars indicating the standard deviation (often invisible due to the range of the error bars being smaller than the plot symbols). The dashed line indicates identical overlap between two samples for both comparisons, which is the expectation if every cell in the sample would have contributed maximally one mRNA molecule (25). The increase of overlap for all sample pairs after redistribution indicates that a substantial fraction of the cells contributed multiple mRNA molecules. Hence, the probability for a given cell to contribute a TCRβ mRNA is expected to be considerably lower than the upper bound shown in (B). [file Image_7.jpeg]
