# Supplementary material for: On the feasibility of using TCR sequencing to follow a vaccination response – lessons learned
Source: Front Immunol. 2023 Jul 13;14:1210168. doi: 10.3389/fimmu.2023.1210168 (PMC10374308; doi:10.3389/fimmu.2023.1210168)
Supplement: Supplementary Table 1 — Donor characteristics. [file Table_1.pdf]

Table S1. Donor characteristics

| Donor ID | Age (years) | Sex | CMV status | Timing follow-up sample | Comments |
|---|---|---|---|---|---|
| 1 | 70 | M | CMV- | 6.5 months | |
| 17 | 73 | F | CMV- | 4.4 months | |
| 72 | 71 | F | CMV- | 6.5 months | |
| 105 | 68 | M | CMV- | 5.5 months | |
| 145 | 69 | F | CMV- | 6.2 months | Sequenced twice |
| 157 | 27 | M | CMV- | 6.9 months | |
| 203 | 38 | F | CMV+ | 7.7 months | |
| 204 | 25 | F | CMV- | 6.0 months | Illumina NextSeq; 3 samples per time point |
| 264 | 27 | F | CMV- | 6.8 months | |
| 273 | 31 | M | CMV- | 5.7 months | |
| 292 | 28 | F | CMV+ | 6.5 months | Illumina NextSeq; 3 samples per time point |
| 310 | 30 | F | CMV- | 5.4 months | |
| 311 | 25 | F | CMV- | 5.5 months | |
